# EV Translational Horizons as Viewed Across the Complex Landscape of Liquid Biopsies

**DOI:** 10.3389/fcell.2021.556837

**Published:** 2021-09-20

**Authors:** Bryce Killingsworth, Joshua A. Welsh, Jennifer C. Jones

**Affiliations:** Translational Nanobiology Section, Laboratory of Pathology, Center for Cancer Research, National Cancer Institute, National Institutes of Health, Bethesda, MD, United States

**Keywords:** liquid biopsies, cell free nucleic acids, extracellular vesicles, exosomes, lipoproteins, nanotechnology, therapeutic, diagnostic

## Abstract

Extracellular Vesicle (EV)-based diagnostic and therapeutic tools are an area of intensive study and substantial promise, but EVs as liquid biopsies have advanced years ahead of EVs as therapeutic tools. EVs are emerging as a promising approach for detecting tumors, evaluating the molecular profiles of known disease, and monitoring treatment responses. Although correlative assays based on liquid biopsies are already having an impact on translational studies and clinical practice, much remains to be learned before these assays will be optimized for clinical correlations, functional biological studies, and therapeutic use. What follows is an overview of current evidence supporting the investigation and use of liquid biopsies, organized by specific liquid biopsy components available for analysis, along with a summary of what challenges must be overcome before these assays will provide functional biological insights into the pathogenesis and treatment of disease. The same challenges must also be overcome before it will be feasible to measure and monitor the dosing, distribution, pharmacokinetics, and delivery of EV therapeutics and their cargo in complex biofluids where EVs and circulate with and are co-isolated with a number of other nanoscale materials, including lipoproteins (LPPs), ribonucleoprotein complexes (RNPs), and cell free nucleic acids (cfNA).

## Introduction

Cells of all types produce a number of secreted or shed macromolecular complexes that can be examined for liquid biopsies. The term “liquid biopsy” here refers to a type of assay that uses a biofluid specimen, rather than fragment of tumor tissue, to test for various molecular biomarkers. Liquid biopsies offer the advantage of interrogating tumor biology without invasive surgical or interventional procedures. Furthermore, liquid biopsies may offer a more comprehensive overview of a tumor’s molecular landscape due to the integrative nature of an assay of biomarkers that are detectable in biofluid samples, as compared to the focused interrogative view that is obtained by a tissue biopsy that views only part of a tumor. The ability of liquid biopsies to represent the full complexity of heterogeneous tumor landscapes is especially relevant when there are multiple metastases.

Just as tissue biopsies can be used to evaluate multiple different types of molecular markers in a tumor, liquid biopsies can also be used to evaluate multiple different types of molecular markers. Biofluid samples may include circulating tumor cells (CTCs), cell-free nucleic acid (cfNA), or extracellular vesicles (EVs) from multiple tissues or infectious agents within the body. The highly heterogenous composition of biofluids must be considered when choosing and considering the results of various types of liquid biopsies. The types of biomarkers available in biofluids are highly diverse and include circulating tumor cells (CTCs), cell free nucleic acids (cfNA), lipoprotein particles (LPP), extracellular vesicles (EVs), and exomeres. The development of increasingly sensitive and accurate analytical tools has enabled a wave of recent studies investigating the role these biomarkers in all aspects of disease detection, pathogenesis, treatment, and monitoring. The complexity of the many facets to be considered in rigorous characterization of EVs from biofluids provides an informative perspective on the complexities that remain to be tackled when considering methods for monitoring delivery, circulation, kinetics, and clearance of EV-based therapeutics.

## Tumor Associated Proteins

Although not commonly referred to as assays of the “liquid biopsy” type, blood tests for proteins such as Prostate Specific Antigen, CA19-9, and CA125 have been used for decades (Gold and Freedman, 1965). These tests detect specific proteins that are more highly expressed by certain tumors than by healthy tissues. In common practice, these basic, well-established clinical assays form the foundation for how clinicians currently consider results from blood tests. The most important considerations with each of these tumor-associated protein markers is that none is perfectly sensitive and specific at all stages of tumor growth and progression. If or when tumors progress, especially in the setting of metastatic disease, overall tumor heterogeneity increases, and a malignancy that was previously positive for one of these markers may devolve and lose marker expression. Each of these markers is limited in utility by the degree to with each of these markers, by virtue of a being a single marker associated with a complex and heterogeneous disease, incompletely reflects the status of the disease. Nonetheless, as benchmarks for the standard of care across decades, these assays are the “liquid biopsy” markers that a new generation of liquid biopsies must improve upon, and these traditional blood tests are the assays against which new liquid biopsy assays are most commonly first compared.

## Circulating Tumor Cells

Circulating tumor cells have the next longest track record in the field of liquid biopsies. Across more than a decade, CTC-based assays have provided valuable insights into tumor heterogeneity and evolution. Because CTCs contain the full cellular composition of a tumor cell, these CTCs provide a means of direct interrogation of disease-associated genetic variants, including genetic variants that inform treatment selection. Such CTC assays, especially assays that combine tests for tumor-associated surface proteins with tests for tumor-specific genetic mutations, have been shown to predict treatment responses and improve treatment selection for several types of cancers (Autio et al., 2014; Dittamore et al., 2014; Scher et al., 2016, 2017; Lack et al., 2017; Figueroa and Carter, 2018; Armstrong et al., 2019; Salami et al., 2019). However, CTCs circulate in low concentrations, typically at levels of 1–10 CTCs per milliliter of blood, and some types of tumors are associated with lower levels of CTCs than others, so, for many types of tumors, it can be difficult to capture a full range of tumor heterogeneity in CTCs from standard blood sample volume (Figueroa and Carter, 2018; Jin et al., 2019).

Although CTCs carry an individually complete “package” of cellular cargo (proteins, nucleic acids, phospholipids, etc.), which reflects the composition of some part of a tumor, extracellular biofluid components are inherently fragmentary. In other words, these biofluid components individually are only partial representations of the cell from whence they originated, but these components also can be analyzed to interrogate the status of the tumor.

## Cell-Free Nucleic Acids, Circulating Tumor DNA

DNA can be detected in the extracellular portion of biofluids, and this cfNA is found in both the biofluid fractions that are unassociated and that are associated with other biofluid components, such as extracellular vesicles. Despite this heterogeneity and the present imprecision of current scientific methods to accurately or consistently catalog the distribution of cfNAs and the molecular carriers of cfNA in biofluids, levels of circulating DNA are consistently found to be higher in the setting of cancers, in a manner that is proportional to the stage of the tumor (Newman et al., 2014). Due to the sensitivity of current techniques, such as cancer personalized profiling (CAPP-seq) deep sequencing, for interrogating DNA profiles, ctDNA assays are not only able to detect advanced disease (Newman et al., 2014; Scherer et al., 2016; Hellmann et al., 2020), but also detect molecular residual disease following treatment delivered with curative intent (Chaudhuri et al., 2017; Azad et al., 2020), thereby enabling selective and informed addition of modifications in patients’ individual treatment plans. Moreover, such ctDNA-based assays have demonstrated an ability to delineate tumor responses at extended intervals after treatments such as immunotherapy in a manner may predict the risk of eventual progression (Hellmann et al., 2020). Moreover, the detection of tumor-specific DNA profiles using similar ctDNA assays has been found to be predictive of responses not only to systemic, but also to localized, ablative treatments with radiation (Phillips et al., 2019).

## Cell-Free Nucleic Acids, Extracellular RNA

**FIGURE 1:**
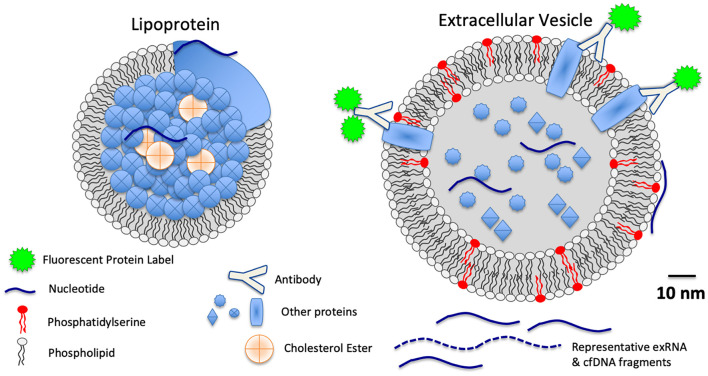
Schematic scale diagram of relative sizes of LPPs, EVs, extracellular RNA (exRNA), cell free DNA (cfDNA), and proteins found in biofluids.

Among the cfNA fraction of circulating biofluids are various extracellular RNA molecules and complexes, collectively referred to as exRNA. Many exRNA molecules, including both long and short RNA, as well as RNA that is either coding or non-coding, are associated with carriers, including EVs and LPPs, that span across a 30 − > 200 nm size range (Figure 1). This size range overlaps and includes some portion of the EVs, LPPs, and exomeres discussed below. Moreover, different methods of RNA isolation along with the wide range of biofluid processing methods that are intended to enrich for specific carrier classes, produce an enormous range of variance related to these different methods (Sadik et al., 2018; Murillo et al., 2019). There exists, therefore, much variability between previously reported studies that report exRNA profiles a with regard to the exact nature of specific carrier fractions, simply because methods previously reported to isolate “exosomal RNA,” have been found to in fact isolate EV-associated RNA molecules together with other RNA-binding co-isolates that are neither exosomal nor EV-associated (Sadik et al., 2018). Nonetheless, some such preparations that were initially understood to be comprised of only exosomes, which in fact have since been found to contain both exosomal and non-exosomal RNA, have been demonstrated to be especially informative for robust tumor detection and discrimination of tumor grade or risk-stratification (McKiernan et al., 2016, 2018). Because of the broad diversity of exRNA forms and carriers that are found in biofluids, liquid biopsies with exRNA is an area of active, rapidly evolving area of investigation where additional advances are anticipated (Li et al., 2018).

## Extracellular Vesicles

Extracellular vesicles (EVs) as biomarkers (and mediators) of disease is an emerging area of research in all medical fields. The term EV refers to a broad range of lipid bilayer membrane-bound vesicles that are either shed from or secreted from cells, and these nanoscale fragments of cells are ubiquitously released from all types of cells, ranging from marine bacteria to human neurons. EVs carry biological information and mediate cell-to-cell effects and extracellular niche formation, but current scientific methods are only beginning to be able to accurately characterize their diversity and compositions (Witwer et al., 2013; Thery et al., 2018; Witwer and Thery, 2019).

Extracellular vesicles are both abundant and highly stable in the peripheral circulation. Estimations of concentrations per milliliter of blood vary widely. Most estimations are on the order of 10–1000-fold more concentrated than the number of red blood cells (RBC) per mL, where an average mL of blood may contain 5 million RBCs, the same volume of blood may contain 10^7^–10^9^ EVs (see Table 1). Because EVs are reported to be highly stable, and because there appears to be rapid turnover of circulating EVs in the reticuloendothelial system, it has been inferred that EVs in circulation are recycled in a time span of hours or days, not weeks as erythrocytes or other cellular components in circulation. The lipid bilayer of EVs protects internal cargo RNA and improves the stability of RNAs for testing in liquid biopsies (Laurent et al., 2015; Srinivasan et al., 2019). The heterogeneity of EVs, including exosomes, oncosomes, apoptotic bodies, among many other subsets, provides a deep reservoir of cellular data that reflects the status of many different cells in many different stages. To parse out this information, EV subset classification has been performed with various methods that separate or classify the EVs according to specific features, including size, density, molecular phenotype (e.g., surface protein expression), source, and functional activity (Witwer et al., 2013; Thery et al., 2018; Witwer and Thery, 2019).

**TABLE 1:** Estimated concentrations of liquid biopsy components per mL blood in cancer.

**Blood component**	**Concentration (per mL)**	**Size**
Red blood cells	5,000,000,000	5–7 microns
White blood cells	7,000,000	7–17 microns
Platelets	300,000,000	2 microns
Circulating tumor cells	0–10	10 microns
ctDNA	5,000,000,000	–
exRNA	10,000,000,000	–
Extracellular vesicles	1,000,000,000	50 nm to microns
Lipoprotein particles	10,000,000,000,000,000	25–120 nm
Exomeres	>1,000,000,000,000	<50 nm

*Updated from Brock et al. (2015) *TCR* 2015.*

The submicron size of EVs is both an advantage and hinderance to the study of EVs as biomarkers. The submicron size of most EVs enables their diffusion from the tumor microenvironment into peripheral biofluids, such as serum, plasma, and urine. Thus, intratumoral “data” can be interrogated in peripheral biofluids. However, EVs overlap in size with other macromolecular assemblies in biofluids, such as lipoprotein particles (LPPs) and ribonucleoprotein (RNP) complexes, so exact biological classification of that EV-associated “data” is non-trivial and must be interpreted cautiously. Analyses of individual EVs is especially challenging, but new methods are being refined for EV profiling at the single EV level (Thery et al., 2018; Morales-Kastresana et al., 2019; Murillo et al., 2019).

Despite the many ambiguities and complexities that must be considered with currently available methods for EV fractionation, several studies indicate that EVs and their cargo are deep reservoirs to mine for biomarker profiles. EVs and their cargo profiles have been shown to reflect tumor stage, molecular classification, prognosis, response to treatment, and risk of recurrence. Early EV studies are also indicating therapeutic and functional roles for EVs, as several studies indicate that EVs contribute to the pathogenesis of tumor progression. PD-L1 expression on tumor-derived EVs is associated with immune evasion (Ricklefs et al., 2018), and in prostate cancer patients, large EVs, or “large oncosomes,” carry most of the ctDNA in plasma (Vagner et al., 2018; Zijlstra and Di Vizio, 2018), and these large prostate cancer oncosomes mediate changes in the tumor microenvironment and prostate fibroblast reprogramming, via AKT1-induced MYC activation (Minciacchi et al., 2017).

## Lipoproteins

Lipoproteins, commonly subdivided into classifications such as LDL, HDL, and chylomicrons, overlap in size with EVs (Figure 1), and they also are comprised of lipids, proteins, and nucleic acids, albeit without the lipid bilayer that defines EV structures. Few studies have specifically interrogated lipoprotein-associated RNA as biomarkers of disease. However, those studies which have investigated LPP exRNAs have found striking correlations between LPP-associated exRNA profiles and disease (Vickers and Moore, 2013; Guo et al., 2015; Michell et al., 2016). The scale and relevance of LPP-associated biomarkers is likely to be significant in the future, as LPP complexes are estimated in serum and plasma at concentrations of the 10^16^ individual LPPs/mL (based on Jens, Circulation Research 2017 121:920–922, roughly 10^7^-fold more concentrated than EVs, for example). Future studies of LPP-associated biomarkers in liquid biopsies is an area for future research that is, to some extent, awaiting improved methods for subdividing relevant LPP populations from other biofluid components.

## Exomeres

Exomeres have emerged as a previously unidentified component in liquid biopsies, but their biogenesis, function, and distribution are not yet well understood. This fraction from serum or plasma can be isolated with size-based fractionation, and identifiable differences between exomeres and LPPs have not been fully identified. The ambiguity in separating the exomere and LPP fractions relates to both the small size of the exomeres (<50 nm), which overlaps with the size of HDLs and some very small EVs, and the lack of defining, robustly segregating markers to reliably distinguish these different components. EVs, lipoproteins, and other co-isolated particles from biofluids are known to bind to or associate with other extracellular materials, such as cfDNA, albumin, and fibronectin, which will may or may not be integral EV components, freely circulating molecules, or detached matrix components. Thus, even “clean” separations of liquid biopsy components are expected to contain “co-isolates,” and high-resolution fractionation based on component type is challenging with currently available instruments and methods.

Newly developed and refined methods, such as asymmetric field flow fractionation, are beginning to produce additional insights into liquid biopsy “co-isolate” compositions. Using asymmetric field flow fractionation, plasma samples have been separated in a manner that shows that not only a diversity of membrane bound vesicles (EVs) and lipoproteins (LPPs), but also an as-of-yet minimally characterized assortment of highly abundant macromolecular complexes comprised of lipid, protein, and nucleic acid assemblies that are not immediately classifiable as lipoproteins, extracellular vesicles, or any other known biofluid component (Zhang et al., 2018; Zhang and Lyden, 2019). Because exomeres lack lipid bilayers and because they are not immediately classifiable as lipoproteins, these biofluid components, therefore, have been termed “exomeres” and the significance of the information carried in their molecular cargo is being actively investigated (Zhang et al., 2018; Zhang and Lyden, 2019).

## Future Directions for Liquid Biopsies

Overall, these various liquid biopsy components are only beginning to be understood in terms of their distributions, their biological functions, and their clinical utility as biomarkers for disease. Because of the manner in which tumors are associated with molecular alterations that may be detected in these components, this is a rapidly expanding area of research. Fortunately, this coincides now with rapidly converging advancements across the fields of nanomedicine, genomics, microfluidics, and engineering, which will improve accuracy, precision, and relevance of liquid biopsy component profiles in the future.

For specific use in the setting of oligo metastatic disease (and in widely metastatic disease), all of the extracellular components delineated above afford the benefit of being able to interrogate molecular profiles that are expected to reflect an integral sampling across heterogenous cellular states. Thus, these sorts of assays may offer significant advantages over focal tumor biopsies that cannot interrogate the range of heterogeneity across all tumor sites in the patient. Thus, these liquid biopsy-based tumor-associated and response- associated molecular signatures are rapidly emerging as robust sources of data to guide diagnosis, treatment selection, and monitoring, with several new assays and ongoing clinical trials.

## Future Directions for EV Therapeutics

Several EV-focused therapeutic solutions have entered development in recent years (Zipkin, 2019). Therapeutic utility of cell-derived EVs presumes that EVs will be able to be manufactured in sufficient quantities, with appropriately quality-controlled purity, reproducibility, and quantifiable dosing. Broadly speaking, the production and necessary quality control considerations for EV therapeutics are very similar to those of biological and cellular therapeutic agents. The regulatory handling of EV therapeutics is overseen by the FDA’s Center for Biologics Evaluation and Research, which recently has issued public safety warnings relating to unapproved EV therapeutics following reports of serious adverse events related to “unapproved products marketed as containing exosomes” (December 9, 2019), stating that, “There are currently no FDA-approved exosome therapeutic products.”

One central barrier to translating EV-therapeutic products to the clinic is the inherent cargo heterogeneity of EVs produced by single cells. Because the full range of EVs across the full range of EV sizes (as small as 30–50 nm) cannot be evaluated as individual vesicles, methods for individual vesicle characterizations are a topic of intensive research and development, which if successful would be of substantial benefit to this field.

A second central challenge for the development of EV-therapeutics is defining the relevant unit(s) of activity. When any therapeutic agent is given to patients, a specific dose to be administered is defined. In protein therapeutics, the dose is defined in units of protein concentration. In cellular therapeutics, the dose is defined in terms of number of cells of a defined type. In EV therapeutics, the measure of “dose” remains to be determined rigorously, since the field lacks instruments to accurately measure EV concentrations and comprehensively characterize EV heterogeneity. Presently, it is feasible to measure bulk payload, for example, in a similar manner to the measurement of drug cargo encapsulated in liposomes in a liposomal preparation; however, qualitative detection or quantitative enumeration of payload contained in few or single EVs is an unmet need.

Studies developing improved methods for measuring EVs and other co-isolates in liquid biopsies will pave the way for the development of qualified and standardized handling, formulation and storage for EV therapeutics. Such challenges are common at frontiers of great promise. Mapping the way forward is an ongoing endeavor with close collaborations across the diagnostic and therapeutic frontiers of EV biology, and despite the great distances ahead, great discoveries and advancements are continuing to be made along the way.

## Author Contributions

All authors contributed to the development, composition, and revision of this manuscript.

## Conflict of Interest

The authors declare that the research was conducted in the absence of any commercial or financial relationships that could be construed as a potential conflict of interest.

## Publisher’s Note

All claims expressed in this article are solely those of the authors and do not necessarily represent those of their affiliated organizations, or those of the publisher, the editors and the reviewers. Any product that may be evaluated in this article, or claim that may be made by its manufacturer, is not guaranteed or endorsed by the publisher.
